# Supplementary material for: The Effect of C/Si Ratio and Fluorine Doping on the Gas Permeation Properties of Pendant-Type and Bridged-Type Organosilica Membranes
Source: Membranes (Basel). 2022 Oct 13;12(10):991. doi: 10.3390/membranes12100991 (PMC9610907; doi:10.3390/membranes12100991)
Supplement: Supplementary file 1 [file membranes-12-00991-s001.zip › membranes-1928892-supplementary.pdf]

membranes

Article

The effect of C/Si ratio and fluorine doping on the gas permeation properties of pendant-type and bridged-type organosilica membranes

Ikram Rana, Takahiro Nagaoka, Hiroki Nagasawa, Toshinori Tsuru and Masakoto Kanezashi*

Hiroshima University, Higashi-Hiroshima, 739-8527, Japan,
* Correspondence: kanezashi@hiroshima-u.ac.jp

Citation: Rana, I.; Nagaoka, T.; Nagasawa, H.; Tsuru, T.; Kanezashi, M. The effect of C/Si ratio and fluorine doping on the gas permeation properties of pendant-type and bridged-type organosilica membranes. *Membranes* **2022**, *12*, 991.https://doi.org/10.3390/membranes12100991

Academic Editor(s): Shigeyuki Uemiya

Received: 6 September 2022
Accepted: 5 October 2022
Published: 13 October 2022

Publisher's Note: MDPI stays neutral with regard to jurisdictional claims in published maps and institutional affiliations.

Copyright: © 2022 by the authors. Licensee MDPI, Basel, Switzerland. This article is an open access article distributed under the terms and conditions of the Creative Commons Attribution (CC BY) license (https://creativecommons.org/licenses/by/4.0/).

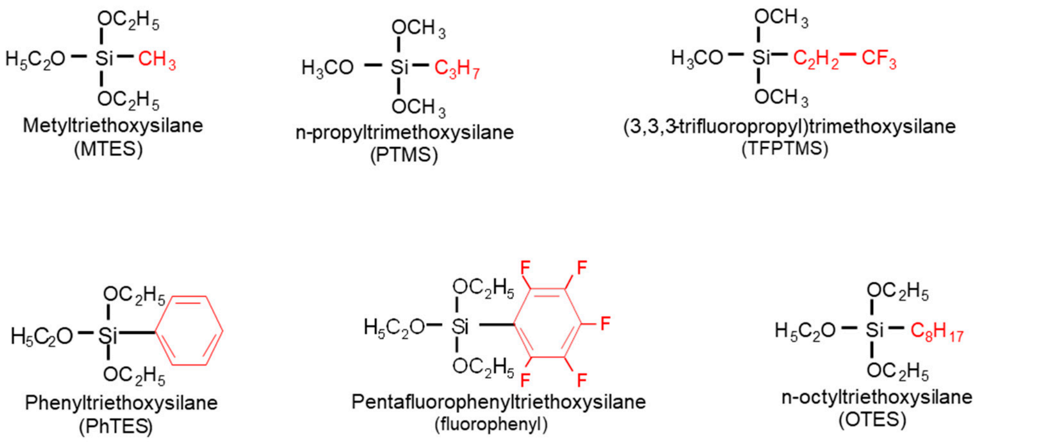

Figure S1. Chemical structures of pendent-type organosilica monomers (C_1-C_8) utilized to fabricate organosilica membranes.

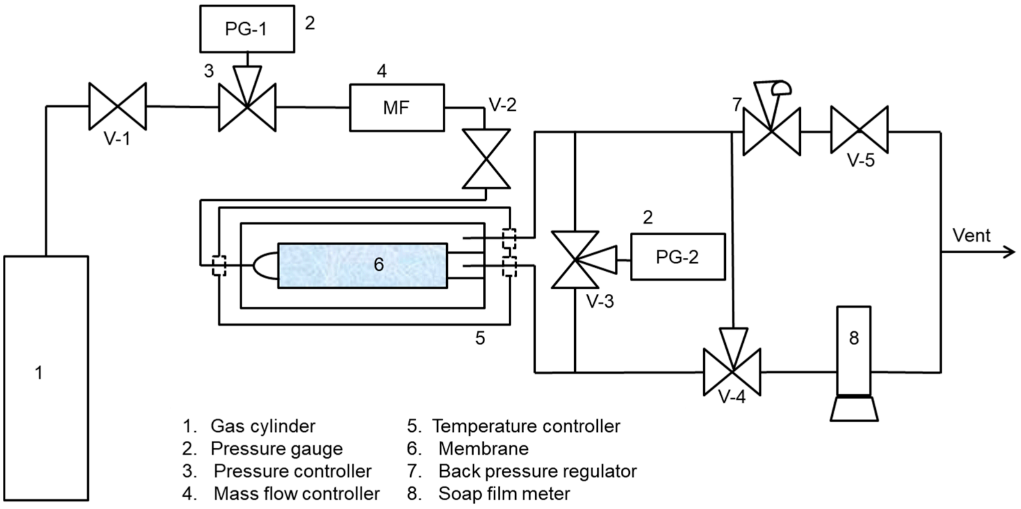

Figure S2. Schematic image of the experimental apparatus used for single-gas permeation measurements.

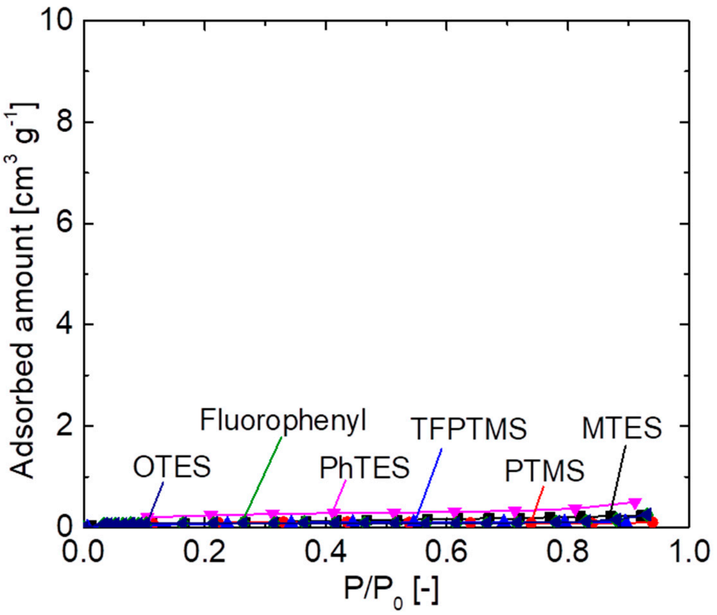

Figure S3. N_2 adsorption isotherms at 77 K for various pendant-type organosilica-derived gels calcined at 300 ⁰C under a N_2 atmosphere.

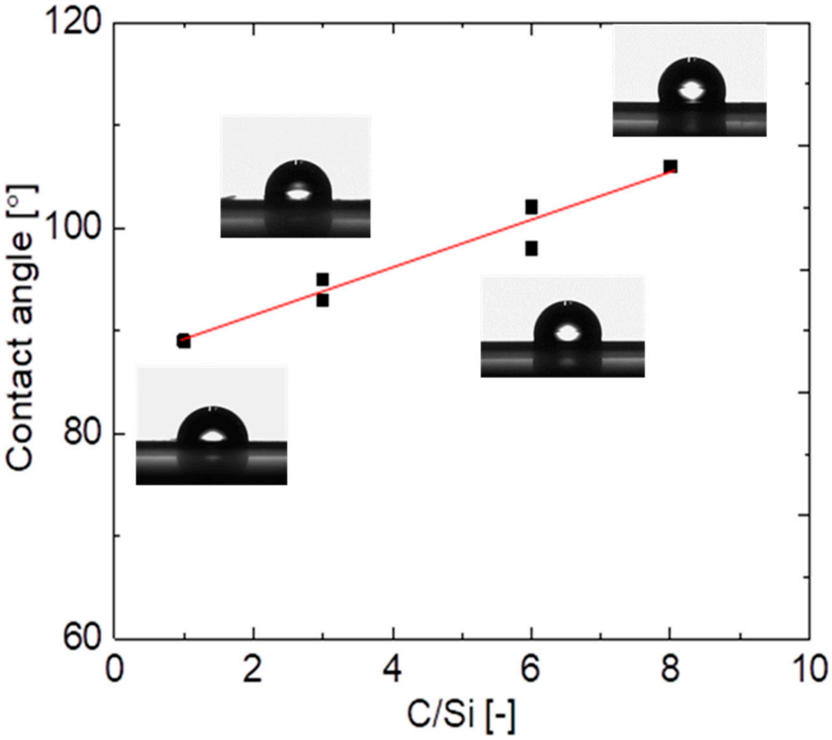

Figure S4. Water contact angle of various pendent-type organosilica films calcined at 300 ⁰C under N_2 atmosphere.

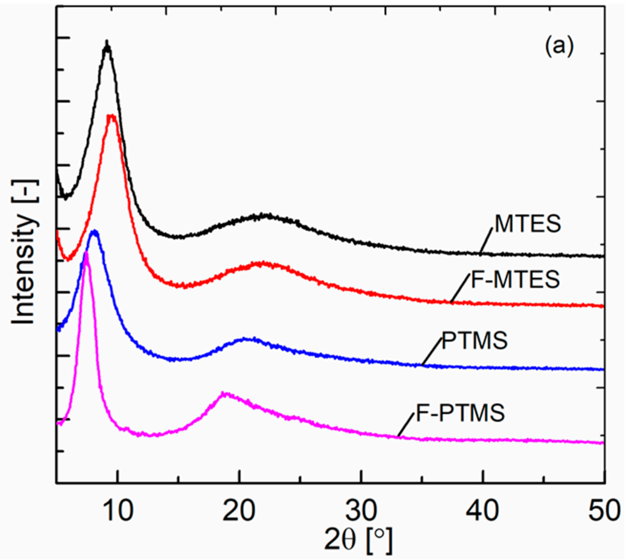

Figure S5. XRD pattern of fluorine-doped and undoped pendent-type organosilica gels calcined at 300 ⁰C under N_2 atmosphere.

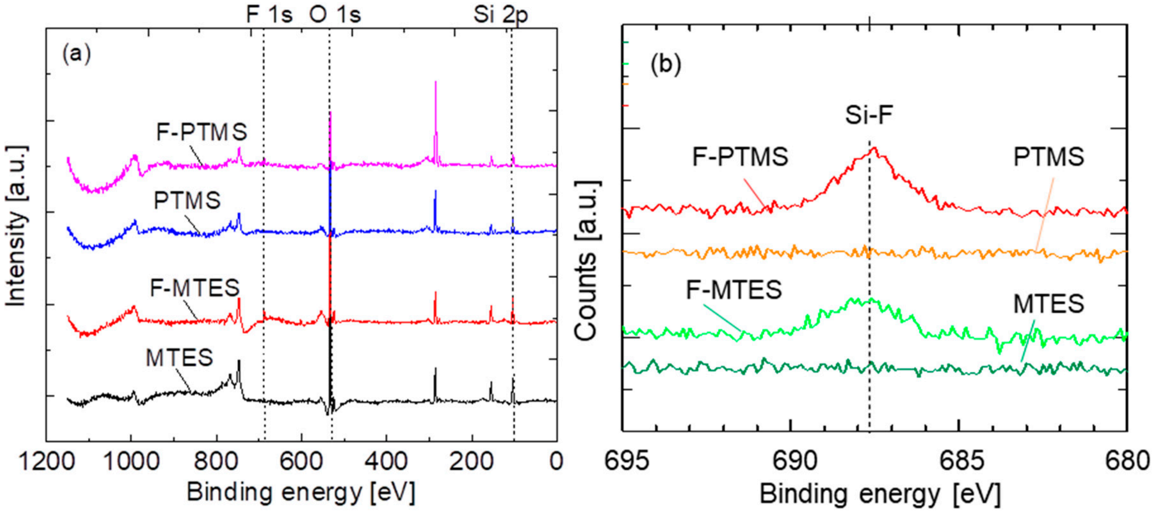

Figure S6. XPS spectra in the range of 0-1200 eV (a) and F 1s spectra (b) for fluorine doped and undoped pendent type organosilica gels calcined at 300 ⁰C under N_2 atmosphere.

Table S1. Si-OH/Si-O-Si peak area ratio for F-doped/undoped pendent organosilica films calcined at 300 ⁰C under N_2 atmosphere.

	Before calcination	After calcination
MTES	0.24	0.005
F-MTES	0.19	0.001
PTMS	0.38	0.007
F-PTMS	0.33	0.003

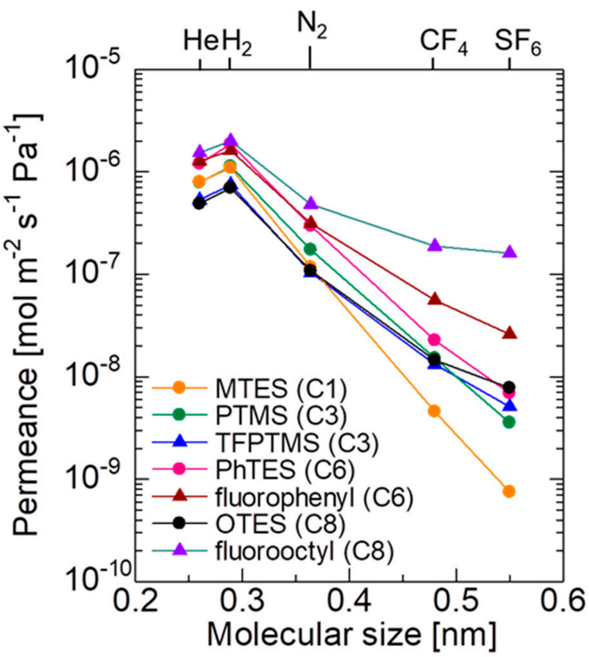

Figure S7. Molecular size dependence of pendant-type (C1-C8) organosilica membranes calcined at 300 ºC under a N_2 atmosphere.

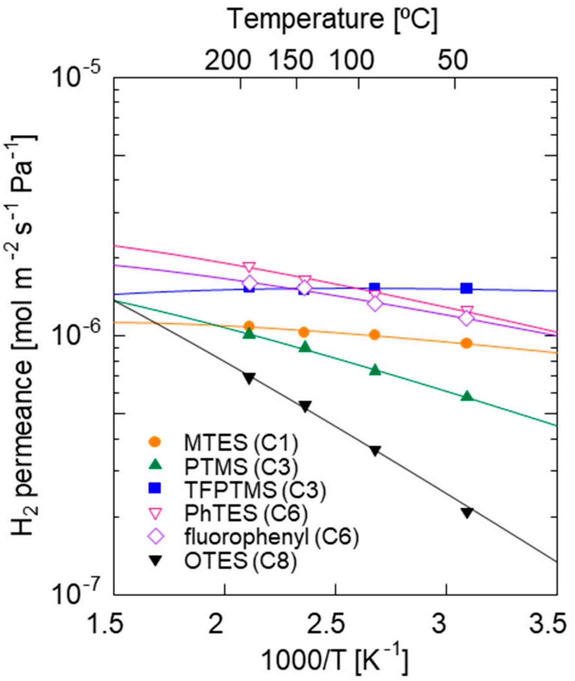

Figure S8. Temperature dependence of H_2 permeance for pendant-type (C1-C8) organosilica membranes calcined at 300 ºC under a N_2 atmosphere.

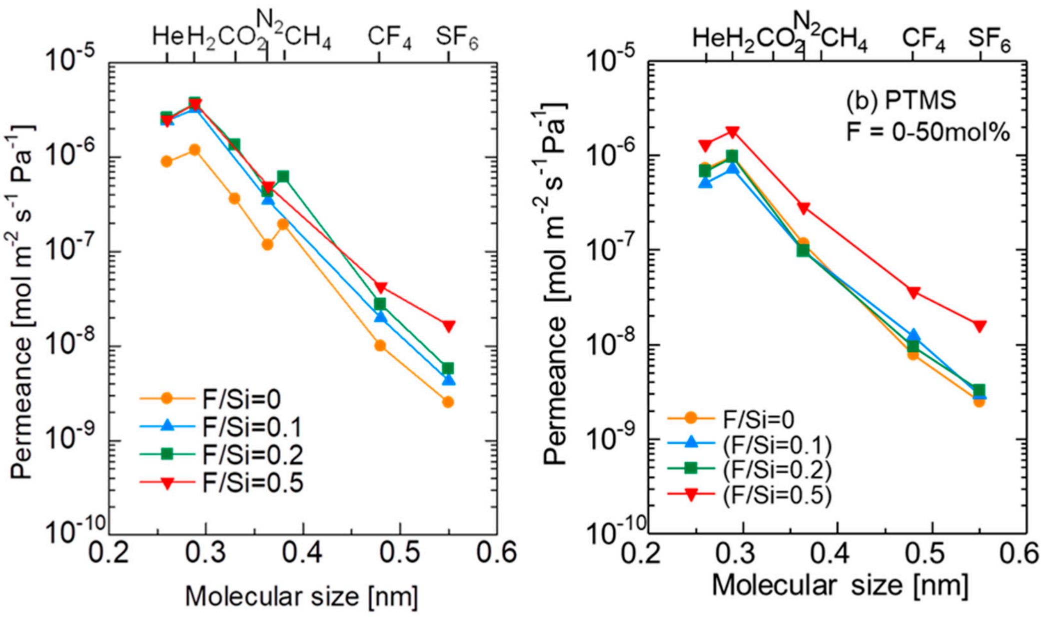

Figure S9. Molecular size dependence for fluorine-doped and undoped MTES (a) and PTMS (b) pendant-type (C_1-C_3) organosilica membranes calcined at 300 ⁰C under a N_2 atmosphere.

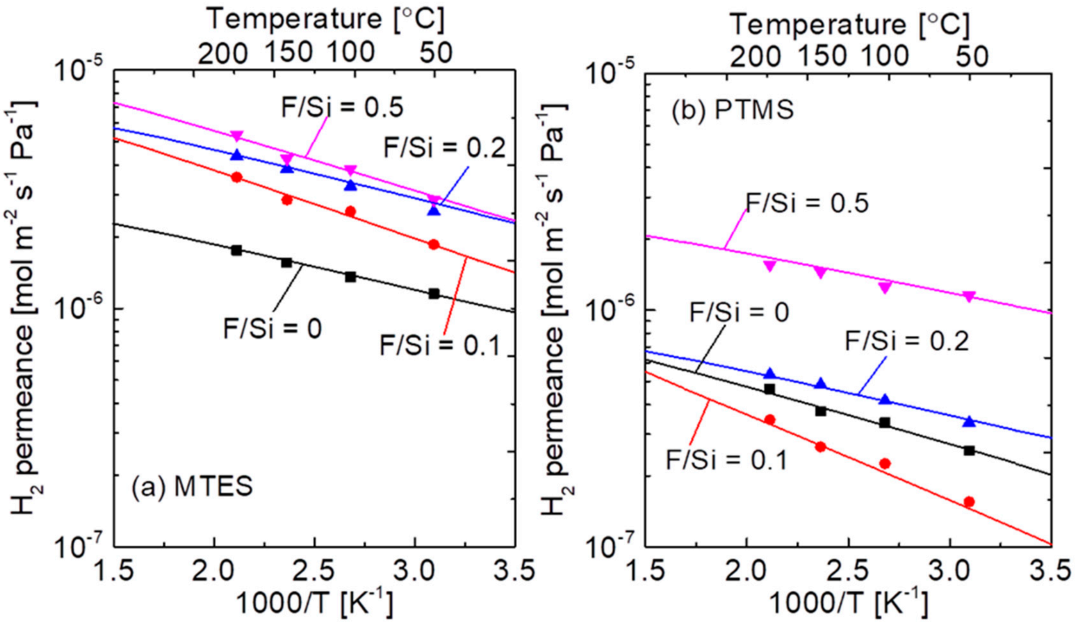

Figure S10. Temperature dependence of H_2 permeance for fluorine-doped and undoped MTES (a) and PTMS (b) pendant-type (C_1-C_3) organosilica membranes calcined at 300 ⁰C under a N_2 atmosphere.
